# Awareness, experiences, and opinions by owners, breeders, show judges, and veterinarians on canine Brachycephalic Obstructive Airway Syndrome (BOAS)

**DOI:** 10.1186/s40575-024-00137-4

**Published:** 2024-03-08

**Authors:** Elina Åsbjer, Åke Hedhammar, Karolina Engdahl

**Affiliations:** 1 Department of Applied Animal Science and Welfare, Swedish Centre for Animal Welfare, Swedish University of Agricultural Sciences, PO Box 7054, 750 07 Uppsala, Sweden; 2 Department of Clinical Sciences, Small Animal Medicine, Swedish University of Agricultural Sciences, PO Box 7054, 750 07 Uppsala, Sweden; 3 Department of Clinical Sciences, Swedish University of Agricultural Sciences, PO Box 7054, 750 07 Uppsala, Sweden

**Keywords:** Dogs, Canine, Brachycephalic Obstructive Airway Syndrome, Survey

## Abstract

**Background:**

Exaggerated brachycephalic features have been highlighted over the last decade by their profound effect on the health and welfare of the affected dogs. The term brachycephalic obstructive airway syndrome (BOAS) was launched in the early 2000s and has received worldwide attention and awareness. At the same time, the popularity of brachycephalic dogs increased. This study aimed to reveal the awareness and experiences of health issues related to the physical appearance of brachycephalic breeds and compare perceptions and opinions on how to counteract these issues by various stakeholders (dog owners, veterinarians, dog breeders, and show judges) by performing an online survey.

**Results:**

Altogether, 1602 owners, 1551 breeders, 118 show judges, and 557 veterinarians participated. Awareness and experiences of conformation-related health issues were common among all stakeholder groups. Most participants agreed fully or partly that health issues related to conformity threaten the health of brachycephalic breeds; that the measures taken so far are positive; and that guidelines on the appearance of a dog should be based on knowledge regarding health issues related to physical appearance. A disagreement was noted on further measures to be taken and the importance of adhering to a breed standard.

**Conclusions:**

All stakeholders were aware of health issues related to the appearance of brachycephalic dogs, but had variable personal experiences of these issues. Most participants agreed fully or partly that health issues related to conformity threaten the health of brachycephalic breeds, and that attention to these issues and measures taken so far are positive. However, there is a disagreement on further actions to be taken and the importance of adhering to a breed standard. These findings could be used to understand and bridge the gap in opinions between stakeholders and to refine methods to influence the health of dogs with exaggerated brachycephalic features.

**Supplementary Information:**

The online version contains supplementary material available at 10.1186/s40575-024-00137-4.

## Plain english summary

Exaggerated anatomical features in dogs, especially related to brachycephaly (short and wide skull and nose), have been highlighted over the last decade by their profound effect on health and welfare. Despite the attention, awareness, and actions to counteract the extent and occurrence of brachycephaly, it is now considered a serious threat to the survival of the most severely affected breeds. However, an ongoing polarisation in public media indicates that there is a disagreement among stakeholders on how these health issues should be handled.

We performed an online survey to reveal the awareness and experiences of health issues related to the physical appearance of dogs with short/wide head and to compare perceptions and opinions on how to counteract these issues among various stakeholders. The survey was distributed to a random sample of owners and breeders of brachycephalic as well as non-brachycephalic dogs, show judges, and veterinarians. Altogether, 1602 owners, 1551 breeders, 118 show judges, and 557 veterinarians participated.

Awareness and experiences of health issues related to brachycephaly were common—but varied depending on personal experiences—in all stakeholder groups. Most participants agreed fully or partly that health issues related to physical appearance threaten the health of brachycephalic breeds, that the measures taken so far are positive, and that guidelines on the appearance of a dog should be based on knowledge of health issues related to physical appearance. A disagreement was noted on further measures to be taken and the importance of adhering to a breed standard.

These findings can be used to understand and bridge the gap in opinions between stakeholders and to refine methods to influence the health in dogs with exaggerated brachycephalic features.

## Background

Exaggerated brachycephalic features have been highlighted over the last decade by their profound effect on health and welfare in dogs [1, 2]. The term brachycephalic obstructive airway syndrome (BOAS) was launched in the early 2000s, followed by a dramatic increase in the popularity of brachycephalic dogs [3, 4]. To counteract the detrimental effects of exaggerated brachycephalic features—especially those causing BOAS – attention, awareness and actions have been instituted. Despite efforts, BOAS is still a significant health and welfare issue worldwide.

Since the first Dog Health Workshop was arranged in Stockholm in 2012, multi-stakeholders with an interest in canine health have met and created the International Partnership for Dogs and DogWellNet, focusing on various aspects of canine health, including extreme conformation [5]. The stakeholders have gathered repeatedly with exaggerated features as one theme [6, 7] and have established an International Collaborative on Extreme Conformation in Dogs (ICEC Dogs) [8]. A textbook with an extensive review and references to activities regarding BOAS was published in 2021 [9].

In Sweden, breed-specific instructions for judges regarding exaggerated features including brachycephaly were introduced in 2009 and a system for veterinarians to report BOAS surgeries to the Swedish Kennel Club (SKC) was established in 2017 [10]. As examples of activities, three conferences focusing on BOAS have been arranged for show judges, breeders, and veterinarians in Sweden since 2016 and an inventory of signs of BOAS in the four focus breeds of this study (English and French bulldogs, pugs and Boston terriers) was performed in 2018 [11]. Based on these activities, the SKC like several national and international welfare and veterinary organisations have made statements and guidelines regarding BOAS [9, 12–14]. These documents conclude that health issues related to BOAS are a serious welfare problem, which may not be recognised by the owners or considered “normal for the breed”, but require immediate action with multi-stakeholder collaboration. Proposed actions include education of show judges, breeders and puppy buyers, examinations of breeding animals, registration of BOAS surgeries, encouraging research, development of objective measures for assessment etc. The guidelines also serve to support practitioners in the diagnosis and treatment of BOAS.

Current research agrees on the health and welfare problems related to short/wide heads and BOAS, and free breathing is a prerequisite for being able to perform many natural behaviours and for having a good quality of life. Despite this, the views on BOAS as a welfare problem differ among stakeholders and strong polarisation in opinions has evolved regarding the extent and severity of BOAS in social media. Although most stakeholders seem to agree that compromised health and clinical signs due to exaggeration of anatomical features must be counteracted, not all seem to agree on the extent of clinical signs of BOAS in brachycephalic dogs and how to handle these health issues [15–17]. Further, most proposed strategies on how to handle BOAS are based on “expert opinions” by just one or two stakeholders [18–20].

This study aimed to explore the awareness and experiences of health issues related to the physical appearance of brachycephalic breeds and to compare perceptions and opinions on how to counteract these issues in various stakeholders (dog owners, veterinarians, dog breeders, and show judges) by performing an online survey. The overall hypothesis was that there is a general agreement among different stakeholders that BOAS is a threat to canine health and welfare, but also a variation in experiences as well as opinions on how to handle these health issues.

## Materials and methods

### Questionnaire development

A questionnaire including questions on the awareness, opinions, and personal experience of health issues related to the physical appearance of brachycephalic dogs was developed within the Swedish Collaborative Committee on Canine Health and Welfare by veterinarians and epidemiologists with experience in health and welfare issues related to BOAS. The Swedish Collaborative Committee on Canine Health and Welfare includes representatives from the Swedish Veterinary Association, the Swedish Board of Agriculture, the County Administrative Board, the Swedish University of Agricultural Sciences, the Swedish Centre for Animal Welfare, and the SKC. A statistician experienced in questionnaire development was consulted during the questionnaire development process. A pretesting including cognitive interviews with read and think-aloud methodology was performed on dog owners [21]. The questions were revised and retested according to the results from the interviews.

The questionnaire included closed questions divided into three parts. A nominal Likert scale with three to five alternatives (depending on the question) was used, and the respondent could choose one response alternative per question. The first part of the questionnaire included questions on the respondents’ background (dog owner, dog breeder, show judge, veterinarian). The second part included eleven questions on the awareness of and opinions regarding health issues related to the physical appearance of brachycephalic dogs, while the third part included specific questions for each stakeholder group regarding personal experiences of owning/breeding/judging/treating brachycephalic breeds with and without clinical signs of BOAS.

The final questionnaire is provided in an English translated version in Supplementary Table 1.

### Brachycephalic breeds

Four focus breeds were identified: the French and English bulldog, pug and Boston terrier. These will further be referred to as focus breeds and compared to dogs of all other breeds.

### Sample size calculation

A sample size calculation was performed on Epitools [22]. A sample size of 408 individuals in each group was required to find differences of ten percentage points between the groups. An error margin of 5% and a power of 80% were used in the sample size calculation.

### Stakeholders and questionnaire distribution

The definition of the stakeholders included in this study is presented in Table 1. The survey was operated online between 26 October 2021 and 29 November 2021. An email including the questionnaire was sent from a professional survey institute (Netigate) and one reminder was sent in case of missing answers. The survey was distributed to:

1. A random sample of dog owners of all breeds except the four focus breeds (*n* = 9571) and a random sample of dog owners of the focus breeds (*n* = 1947). The samples were drawn from the state-operated dog owner registry.
2. A random sample of breeders of all breeds except the four focus breeds (*n* = 4924) and a random sample of breeders of the focus breeds (*n* = 557). The samples were drawn from the SKC’s registry.
3. All registered Swedish show judges (*n* = 259).
4. All veterinarians registered by the Swedish Veterinary Association (*n* = 2858), accounting for 2/3 of all practising veterinarians in Sweden, via a link in the newsletter from the Swedish Veterinary Association.

**Table 1:** Definition of the stakeholders included in a study assessing the awareness, experiences, and opinions on canine brachycephalic obstructive airway syndrome in Sweden

Stakeholder group	
Dog owner	
Of a focus^a^ breed	+/- with dogs registered by the Swedish Kennel Club
Of any other breed	+/- with dogs registered by the Swedish Kennel Club
Dog breeder	
Of a focus^a^ breed	
Of any other breed	
Show judge	
Of a﻿ focus^a^ breed	
Of any other breed	
Veterinarian	Members of the Swedish Veterinary Association

^a^The focus breeds include the French and English bulldog, pug, and Boston terrier

Classification of the stakeholders was possible by source and batch of e-mail addresses as well as by demographic information supplied within the survey. All e-mail addresses were removed before data transfer from Netigate to guarantee the anonymization of respondents.

### Statistical analysis

The statistical analysis was performed in R version 4.2.1 [23]. Incomplete questionnaires were excluded. Categorical variables are presented as numbers and percentages per category. Associations between questionnaire responses and stakeholder groups were evaluated with the Chi-square test and Fisher’s exact test. P-values below 0.05 were considered statistically significant.

## Results

### Classification of stakeholders

**Fig. 1:**
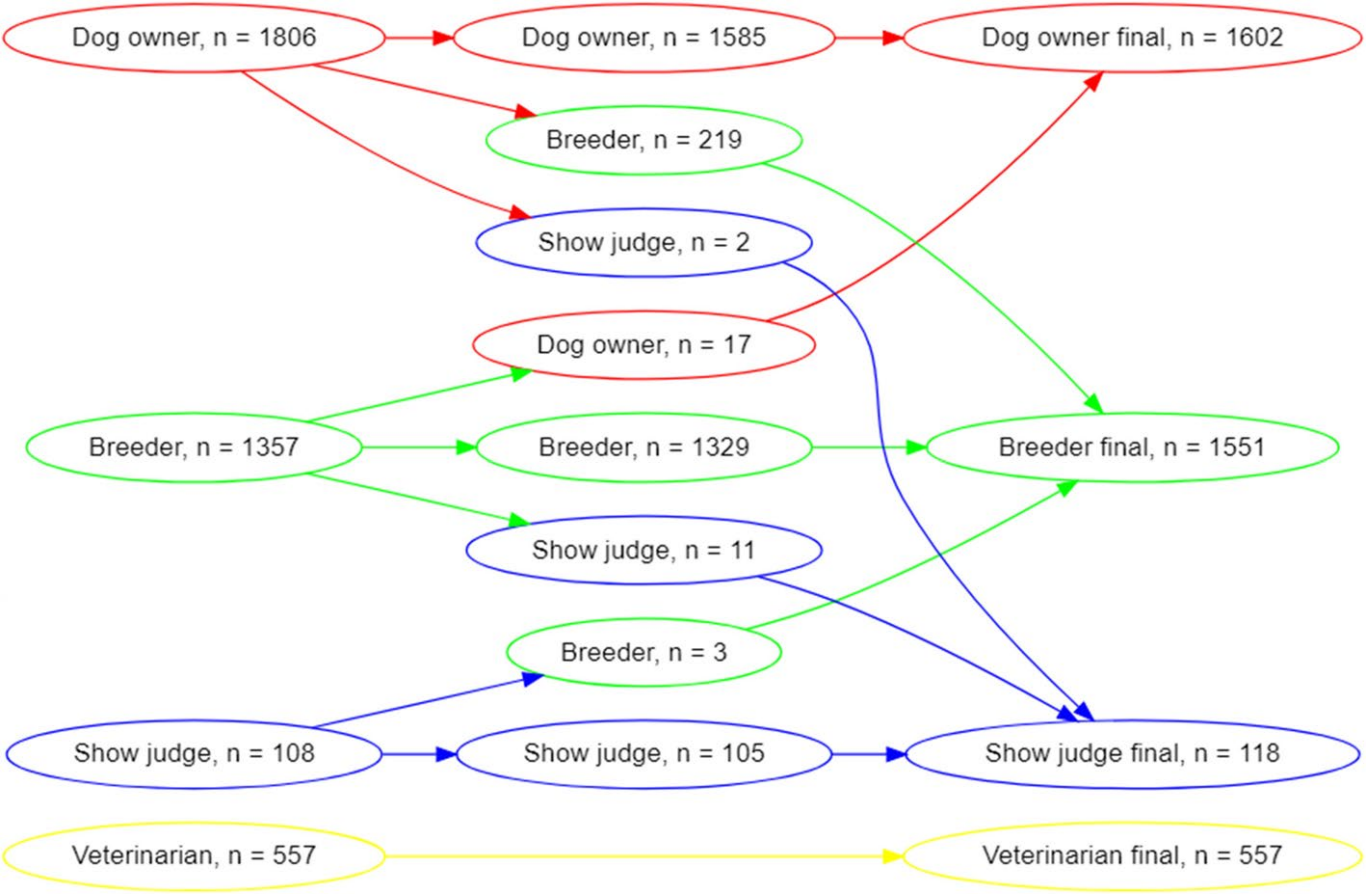
Generation of stakeholder groups, based on the e-mail distribution lists (to the left) and modified according to demographic information included in the questionnaire, resulting in final stakeholder groups (to the right)

A total of 20,116 questionnaires were distributed and 4668 responses were received. In total, 840 questionnaires were incomplete and thus excluded, leaving 3828 questionnaires for further analysis. The response rate was 23.2% before the exclusion of incomplete questionnaires and 19.0% after exclusion. The study population at this stage included 1806 (41.2%) dog owners, 1357 (35.4%) breeders, 108 (2.82%) show judges, and 557 (14.6%) veterinarians, and the response rate of complete questionnaires per group was 15.7% for dog owners, 24.8% for breeders, 41.7% for show judges, and 19.5% for veterinarians. Some respondents were moved from one stakeholder group to another based on their replies to the demographic questions of the questionnaire, which is described in Fig. 1 (e.g. some dog owners indicated that they also were breeders etc.). As several respondents belonged to more than one stakeholder group, the following classification was made: veterinarian (could also be show judge, dog breeder, dog owner), show judge (could also be a dog breeder and dog owner), dog breeder (could also be dog owner), and dog owner (Table 2). The final study population included 1602 (41.8%) dog owners, 1551 (40.5%) breeders, 118 (3.1%) show judges, and 557 (14.6%) veterinarians. Of the dog owners, 465 (29.0%) owned one of the focus breeds, and 137 (8.8%) of the breeders were breeders of the focus breeds. In total, 1029 (64.2%) of the dog owners reported having their dog/s registered in the SKC.

**Table 2:** The number of participants in each stakeholder group in a study assessing the awareness, experiences, and opinions on canine brachycephalic obstructive airway syndrome in Sweden

Stakeholder group	Show judge	Dog breeder	Dog owner
Dog owner, *n* = 1602	-	-	-
Dog breeder, *n* = 1551	-	-	1543 (99.5%)
Show judge, *n* = 118	-	114 (96.6%)	110 (93.2%)
Veterinarian, *n* = 557	0 (0%)	70 (12.6%)	396 (71.1%)

### Awareness of health issues related to the physical appearance of brachycephalic breeds

Almost all respondents replied that they were aware of health issues related to the appearance of short-nosed dogs (Table 3, Q1). Veterinarians were most aware and dog owners least aware: nearly all veterinarians (98.4%) replied that they had heard a lot about the health issues, while the corresponding percentage in the dog owner group was 69.5%. A few respondents, 27 (1.69%) dog owners and 16 (1.03%) dog breeders replied that they were unaware of the issues. Almost all veterinarians (98.4%) had seen or met at least one short-nosed dog with health issues linked to their appearance, while the corresponding percentage in the other stakeholder groups varied between 70.2–78.5% (Table 3, Q2). In total, 337 (60.5%) of the veterinarians experienced that the occurrence of health issues related to the physical appearance of brachycephalic breeds had increased, while 11 (1.97%) experienced that the occurrence had decreased (Table 3, Q3). Of the show judges, 24 (20.3%) experienced an increased occurrence while 38 (32.3%) experienced a decrease.

**Table 3:** Questionnaire responses for the four stakeholder groups regarding the awareness of health issues related to the physical appearance of brachycephalic breeds

	Veterinarian (*n* = 557)	Show judge (*n* = 118)	Breeder (*n* = 1551)	Dog owner (*n* = 1602)
Q1. I am aware of health issues related to the physical appearance of dogs with short noses*				
Yes, a lot	548 (98.4%)	100 (84.7%)	1306 (84.2%)	1113 (69.5%)
Yes, a little	9 (1.62%)	18 (15.3%)	229 (14.8%)	462 (28.8%)
No	0 (0%)	0 (0%)	16 (1.03%)	27 (1.69%)
Q2. I have seen/met dogs with short noses who had health issues related to their physical appearance*				
Yes, several	543 (97.5%)	68 (57.6%)	1028 (66.3%)	861 (53.7%)
Yes, one	5 (0.90%)	21 (17.8%)	189 (12.2%)	263 (16.4%)
No	7 (1.26%)	26 (22.0%)	237 (15.3%)	304 (19.0%)
I do not know	2 (0.36%)	3 (2.54%)	97 (6.25%)	174 (10.9%)
Q3. My experience is that health issues related to the physical appearance of dogs with short noses have*				
Increased	337 (60.5%)	24 (20.3%)	601 (38.7%)	474 (29.6%)
Not change	123 (22.1%)	41 (34.7%)	409 (26.4%)	343 (21.4%)
Decreased	11 (1.97%)	38 (32.2%)	147 (9.5%)	96 (5.99%)
I do not know	86 (15.4%)	12 (10.2%)	385 (24.8%)	680 (42.4%)
I do not agree that such health issues exist	0 (0%)	3 (2.54%)	9 (0.58%)	9 (0.56%)

*P < 0.05 for intergroup comparisons (Chi-squared test or Fisher’s exact test)

### Opinions on how to handle health issues related to the physical appearance of brachycephalic breeds

Opinions by different stakeholders are presented in Table 4. Most respondents in all stakeholder groups agreed fully or partly that health issues seriously threaten the health of brachycephalic dogs (Q4). Over 90% of the respondents in each stakeholder group were fully or partially positive about the measures taken so far to prevent the health issues (Q6). A clear majority (77.0–98.0%) of the veterinarians, breeders, and dog owners were fully or partially positive to further measures to prevent the health issues, while 0.18–11.0% disagreed with further measures (Q7). Of the show judges, 49.2% were fully or partially positive to further measures, while 39.8% disagreed to further measures.

**Table 4:** Questionnaire responses for the four stakeholder groups regarding their opinions on how to address health issues related to the physical appearance of brachycephalic breeds.

	Veterinarian (*n* = 557)	Show judge (*n* = 118)	Breeder (*n* = 1551)	Dog owner (*n* = 1602)
Q4. I think that health issues related to the physical appearance of short-nosed dogs are a serious threat to their health*				
Yes	530 (95.1%)	31 (26.3%)	949 (61.2%)	862 (53.8%)
Partly	25 (4.5%)	59 (50.0%)	428 (27.6%)	462 (28.8%)
No	2 (0.4%)	25 (21.2%)	93 (6.0%)	86 (5.4%)
I do not know	0 (0%)	3 (2.5%)	81 (5.2%)	192 (2.0%)
Q5. I think it is good that the impact of anatomical features on the health of short-nosed dogs is brought to the attention*				
Yes	547 (98.8%)	58 (49.2%)	1253 (80.8%)	1308 (81.6%)
Partly	10 (1.8%)	45 (38.1%)	220 (14.2%)	176 (11.0%)
No	0 (0%)	4 (3.4%)	17 (1.1%)	16 (1.0%)
I do not agree that such health issues exist	0 (0%)	8 (6.8%)	37 (2.4%)	33 (2.1%)
I do not know	0 (0%)	3 (2.5%)	24 (1.5%)	69 (4.3%)
Q6. I think it is positive that measures have been taken to prevent the impact of anatomical features on the health of short-nosed dogs (registration of surgical procedures, guidelines for evaluation at dog shows, certificate of breathing capacity)*				
Yes	516 (92.6%)	70 (59.3%)	1271 (81.9%)	1320 (82.4%)
Partly	32 (5.7%)	38 (32.2%)	193 (12.4%)	130 (8.1%)
No	2 (0.4%)	8 (6.8%)	43 (2.8%)	25 (1.6%)
I do not know	7 (1.2%)	2 (1.7%)	44 (2.8%)	127 (7.9%)
Q7. I think further measures should be taken to prevent the impact of anatomical features on the health of short-nosed dogs*				
Yes	531 (95.3%)	22 (18.6%)	876 (56.5%)	1010 (63.0%)
Partly	15 (2.7%)	36 (30.5%)	319 (20.6%)	257 (16.0%)
No	1 (0.2%)	47 (39.8%)	171 (11.0%)	69 (4.3%)
I do not know	10 (1.8%)	13 (11.0%)	185 (11.9)%	266 (16.6%)
Q8. I am positive to a ban on breeding from dogs with such short noses that it increases the risk of health issues (such as breathing problems, impaired regulation of heat, eye injuries, skin problems)*				
Yes	527 (94.6%)	27 (22.9%)	1017 (65.6%)	1170 (73.0%)
Partly	24 (4.3%)	28 (23.7%)	294 (19.0%)	221 (13.8%)
No	6 (1.1%)	58 (49.2%)	194 (12.5%)	130 (8.1%)
I do not know	0 (0%)	5 (4.2%)	46 (3.0%)	81 (5.1%)
Q9. I am positive to a ban on all breeding of short-nosed dog types or breeds*				
Yes	207 (37.2%)	1 (0.8%)	229 (14.8%)	311 (19.4%)
Partly	216 (38.8%)	9 (7.6%)	335 (21.6%)	405 (25.3%)
No	121 (21.7%)	106 (89.8%)	899 (58.0%)	725 (45.3%)
I do not know	13 (2.3%)	2 (1.7%)	88 (5.7%)	161 (10.0%)
Q10. I think it is important to follow the breed standard from the breed’s country of origin when choosing breeding animals, even if it can be associated with health issues related to their physical appearance*				
Yes	23 (4.1%)	33 (28.0%)	257 (16.6%)	232 (14.5%)
Partly	18 (3.2%)	37 (31.4%)	331 (21.3%)	208 (13.0%)
No	508 (91.2%)	38 (32.2%)	861 (55.5%)	914 (57.1%)
I do not know	8 (1.4%)	10 (8.5%)	102 (6.6%)	248 (15.5%)
Q11. I think that guidelines for how short-nosed breeds should look, ought to be decided based on knowledge regarding health issues related to the physical appearance*				
Yes	543 (97.5%)	55 (46.6%)	1272 (82.0%)	1383 (86.3%)
Partly	13 (2.3%)	53 (44.9%)	240 (15.5%)	188 (11.7%)
No	1 (0.2%)	10 (8.5%)	39 (2.5%)	31 (1.9%)

*P < 0.05 for intergroup comparisons (Chi-squared test or Fisher’s exact test)

All stakeholders except show judges were mostly in favour of banning breeding with individual dogs with a short nose predisposed to clinical signs (Q8). Of the veterinarians, 75.9% were positive or partially positive of banning all breeding with brachycephalic dogs. The corresponding percentage of dog owners, breeders, and show judges that agreed fully or partially to such a ban was 44.7%, 36.4%, and 8.5%, respectively (Q9).

The majority of show judges agreed fully or partly that it is essential to follow the breed standard even if it can be associated with health issues related to their physical appearance, while all other stakeholders mostly disagreed (Q10). A great majority of all stakeholders agreed that guidelines (e.g. breed standards) on the appearance of dogs should be based on knowledge about the relationship between health and conformity (Q11).

In total, 84.4% of the French bulldog owners, 81.2% of the Boston terrier owners, 68.4% of pug owners, and 67.1% of English bulldog owners agreed fully or partially that these problems threaten the dogs’ health (Supplementary Table 2, Q4). The owners of French bulldogs and Boston terriers were also the ones being most positive to further measures and individual bans on breeding (Supplementary Table 2, Q7-Q8).

Opinions and experiences of show judges who judged brachycephalic breeds (BSJ) during 2019 (*n* = 73) and show judges who judged non-brachycephalic breeds (NBSJ) the same year (*n* = 45) are presented in Supplementary Table 3. The percentage of BSJ and NBSJ that had a perception of a decreased prevalence of health issues related to the dog’s conformation was 39.7% and 20.0%, respectively (Q3). Further, the percentage of BSJ and NBSJ that disagreed that such health issues threaten these dogs’ health was 27.4% vs. 11.1% (Q4).

A comparison of owners of dogs of the focus breeds who own/have owned a dog with clinical signs related to their conformation (*n* = 152), with those who have not (*n* = 313) is presented in Supplementary Table 4. Of those with dogs showing clinical sigs, 90.1% agreed that health issues related to conformation are a serious threat to the dogs’ health (Q4). The corresponding percentage in the other group was 69.3%. Further, the percentages of owners in favour of further measures were 84.3% vs. 67.7% in the two groups, respectively, (Q7). In total, 88.2% and 68.7% of owners in the groups agreed fully or partly to banning dogs with such short noses (Q8).

Of the breeders of the focus breeds, 57.7% were positive to further actions (Supplementary Table 5, Q7), and 54.7% were in favour of banning individual dogs from breeding (Q8). The corresponding percentages among breeders of non-brachycephalic breeds were 78.9% and 87.5%, respectively.

### Personal experience of clinical signs related to the appearance of brachycephalic dogs

Questions regarding the personal experiences of clinical signs related to the appearance of brachycephalic dogs were answered by owners (*n* = 465) and breeders (*n* = 137) of the focus breeds, BSJ that assessed brachycephalic dogs during 2019 (*n* = 62), and veterinarians that treated brachycephalic dogs during 2019 (*n* = 423). In total, 152/465 (32.7%) of the owners responded that they had owned a dog with such health issues, and 34/137 (24.8%) of the breeders responded that at least one of the dogs from their breeding was affected (Table 5). Further, 401/423 (94.8%) of the veterinarians responded that they had diagnosed or treated at least one brachycephalic dog with such health issues, while 17/62 (27.4%) of the show judges responded that they had assessed at least one affected dog. Affected breathing was the most common clinical sign reported in all stakeholder groups. Skin or eye problems were reported by 94.8% of the veterinarians, 32.4% of the breeders and 62.5% of the owners. In total, 59.1% of the veterinarians reported that they had euthanized at least one brachycephalic dog for health issues related to the dog’s physical appearance, and 36.2% reported that they had euthanized many dogs for such issues.

**Table 5:** Personal experiences of health issues related to the physical appearance of brachycephalic breeds in different stakeholders who have either diagnosed/treated (veterinarians), judged (show judges), bred (breeders), or owned brachycephalic breeds with such health issues

	Veterinarian (*n* = 401)	Show judge (*n* = 17)	Breeder (*n* = 34)	Dog owner (*n* = 152)
Q21. Has the dog(s) had affected breathing?				
Yes many	385 (96.0%)	9 (52.9%)	8 (23.5%)	11 (7.24%)
Yes one	11 (2.74%)	4 (23.5%)	23 (67.4%)	117 (77.0%)
No	5 (1.25%)	4 (23.5%)	3 (8.82%)	24 (15.8%)
I do not know	0 (0%)	0 (0%)	0 (0%)	0 (0%)
Q22. Has the dog(s) had an affected ability to regulate heat (i.e. overheating in hot weather)?				
Yes many	331 (82.5%)	4 (23.5%)	9 (26.5%)	12 (7.89%)
Yes one	26 (6.48%)	5 (29.4%)	11 (32.4%)	93 (61.2%)
No	29 (7.23%)	5 (29.4%)	11 (32.4%)	43 (28.3%)
I do not know	0 (0%)	3 (17.6%)	3 (8.82%)	4 (2.63%)
Missing	15 (3.74%)	0 (0%)	0 (0%)	0 (0%)
Q23. Has the dog(s) had skin or eye problems?				
Yes many	373 (93.0%)	4 (23.5%)	3 (8.82%)	16 (10.5%)
Yes one	7 (1.75%)	2 (11.8%)	8 (23.5%)	79 (52.0%)
No	5 (1.25%)	10 (58.8%)	23 (67.4%)	57 (37.5%)
I do not know	0 (0%)	1 (5.88%)	0 (0%)	0 (0%)
Missing	16 (4.0%)	0 (0%)	0 (0%)	0 (0%)
Q24. Were the health issues surgically treated?				
Yes many	244 (60.8%)	-	4 (11.8%)	6 (3.95%)
Yes one	52 (13.0%)	-	16 (47.1%)	58 (38.2%)
No	105 (26.2%)	-	12 (35.3%)	84 (55.3%)
I do not know	0 (0%)	-	1 (2.94%)	0 (0%)
Missing	0 (0%)	-	1 (2.94%)	4 (2.63%)
Q25. Did the health issues result in death/euthanasia?				
Yes many	145 (36.2%)	-	1 (2.94%)	2 (1.32%)
Yes one	92 (22.9%)	-	7 (20.6%)	11 (7.24%)
No	164 (40.9%)	-	25 (73.5%)	134 (88.2%)
I do not know	0 (0%)	-	0 (0%)	1 (0.66%)
Missing	0 (0%)	-	1 (2.94%)	4 (2.63%)

## Discussion

Despite the contradictory opinions regarding BOAS-related health issues and how to handle these expressed in social media, no published studies have focused on the disagreements and polarisation between various stakeholders. This study aimed to explore the awareness and experiences of such health issues in dog owners, veterinarians, dog breeders, and show judges. Our survey did target a large fraction of Swedish dog owners, breeders, show judges, and veterinarians. The results verify a wide awareness regarding health issues related to the appearance of brachycephalic dogs, but variable personal experiences and opinions on how to handle these issues.

### Awareness of health issues related to the physical appearance of brachycephalic breeds

The majority of the respondents in all stakeholders in the current study were well aware of the health issues related to the appearance of brachycephalic dogs, which gives a good base for the analyses and validity of the responses to further questions.

No studies have been able to monitor changes in the prevalence of health issues related to brachycephaly in dogs over time. The veterinarians in this study had a perception of an increasing prevalence of such health issues. This could partly be explained by an increasing number of brachycephalic dogs in total, affecting the number of owners of brachycephalic dogs seeking veterinary care for their dogs’ BOAS-related problems. This also indicates that the measures taken to decrease the number of dogs with BOAS-related health issues so far may not have been efficient enough, even though time trends of the actual prevalence of these issues cannot be evaluated in this study. The show judges had a perception of a decreasing prevalence, which could be due to a decreasing proportion of clinically affected dogs participating in dog shows. One-third of the owners of brachycephalic breeds and one-quarter of the breeders had experience from their own dogs or dogs from their breeding being affected by health issues related to their conformation. Even if these numbers are no estimates of prevalence, they indicate that BOAS-related problems are common.

### Opinions on how to handle health issues related to the physical appearance of brachycephalic breeds

The majority of all stakeholders agreed that health issues related to the conformation threaten the health of brachycephalic breeds, that it is good that these issues are brought to attention, and that the measures taken so far are positive, even though the proportion of yes and partly replies varied between 75–100%. However, the view on which clinical signs that are considered a health problem may vary between and within the stakeholder groups. The owners of French bulldogs and Boston terriers agreed most that these health issues seriously threaten the dogs’ health, even though research has shown that pugs have the highest prevalence of BOAS and that pugs, French bulldogs and English bulldogs are the three breeds with highest risk of developing BOAS [1]. Almost a third of the BSJ disagreed that health issues related to the dogs’ conformations pose a threat to the dogs’ health. This may be explained by the normalisation of clinical signs among some owners and BSJ, i.e. the clinical signs of BOAS are considered normal for the breed instead of abnormal for the dog as a species, which has been reported among dog owners in previous research [24, 25], It might also be explained by differences in perception of the extent to which affected breathing and other health issues impact the natural behaviours, welfare, general health, and mental state of the dog. This may in turn affect the views on or agreements to further measures.

The majority of veterinarians, breeders, and owners were fully or partly in favour of banning the breeding of individual dogs with such short skulls that it increased the risk of health issues. This sort of ban already exists in some countries, such as the Netherlands and Norway, in the newly adopted Finnish Animal Welfare Act as well as in the Swedish Board of Agriculture’s regulations for the keeping of dogs and cats in Sweden (SJVFS 2020:8) [26–29], stating that it is prohibited to breed animals in a way that causes suffering. Further, the parent animals are required to have suitable anatomical and physiological features and be free from disease, defects or other features that can be inherited and may cause suffering in the offspring. Despite this, parent animals with anatomical features causing BOAS that may be inherited are used for breeding. Banning *all* breeding of brachycephalic dogs was supported fully or partially by 75.9% of the veterinarians, 44.7% of the owners, 36.5% of the breeders, and 8.5% of the show judges. The veterinarians’ positive view on banning all breeding of brachycephalic breeds could have several explanations. Veterinarians in clinical practice are continuously exposed to brachycephalic dogs with BOAS-related health issues and have knowledge and experience from other health issues related to these breeds' conformity and genetics, such as eye diseases, skin problems, glioma, gastrointestinal problems, and malformation of the vertebral spine [16, 30–34]. A comparison between BSJ and NBSJ showed that the opinion on current and further measures to be taken as well as on banning breeding and adhering to the breed standard varies within the stakeholder group of show judges, indicating an acceptance “of normal for the breed “ by those judging brachycephalic breeds at shows. A majority of BSJ were against further measures while a majority of NBSJ were positive or partly positive to further measures. This adds to the picture of how representatives for different stakeholders have diverse views, opinions, and experiences on the brachycephalic breeds and their health and welfare problems.

The differences in perception are also mirrored by different opinions regarding further actions among breeders of brachycephalic compared to breeders of non-brachycephalic breeds. In total, 35.8% of the breeders of brachycephalic breeds disagreed to further measures to be taken, compared to 8.6% of the breeders of non-brachycephalic breeds. This could be explained by several factors. If the health issues are not considered a problem, then there is no need for further action. This is supported by the fact that 90.1% of the owners of dogs with clinical problems consider these issues as a threat to the dogs’ health compared to 69.3% of the owners of dogs without clinical problems. Further, 84.2% of the owners of dogs with clinical signs were in favour of further measures, compared to 67.7% in the other group. Hence, self-perceived experiences of having a dog suffering from BOAS-related problems seem to affect the willingness to counteract these problems.

The connection between extreme conformation and the risk of BOAS and other health and welfare issues has been reported in several studies [1, 2, 35]. A great majority of all stakeholders agreed that guidelines on the appearance of dogs should be based on knowledge about health and conformity. Despite this, the majority of the show judges (59.3%) agreed (totally or partly) that it is essential to follow the breed standard, even if it can be associated with health issues. The corresponding percentages of veterinarians, breeders, and owners were 7.4%, 37.9%, and 27.5%, respectively. Apart from the fact that show judges may see a healthier fraction of the brachycephalic breeds, this may also be explained by the way show judges interpret breed standards. Breed standards are descriptions of anatomic features of a breed and are “prescribed” by the cynological organisations (Federation Cynologic Internationale (FCI), the Royal Kennel Club (UK) and the American Kennel Club (AKC)). Since the nineteenth century, several standards have been adopted by the Royal Kennel Club [36] and somewhat later similar standards were internationally recognized by the FCI and the AKC.

The breed standards often describe an “ideal picture” of a breed, rather than a range, and are formulated as short, long, small or great. These undefined measures introduce a possibility of subjective interpretations of the breed standards and may lead to promotion of anatomical exaggerations. Breed standards have been in focus since 1960 for their detrimental effect due to the risk of encouraging exaggeration of anatomical features such as too much skin, too short legs or too short and broad skull [37, 38]. Since then, the standards have been rephrased, and a statement saying that”*Only functionally and clinically healthy dogs, with breed typical conformation should be used for breeding”* has been added to breed standards. Further, Breed Specific Instructions (BSI) regarding anatomical exaggerations in pedigree dogs have been instituted worldwide [10]. Despite these measures, there is still a risk of misinterpretation of the breed standards by vague formulations.

### Personal experience of clinical signs related to the appearance of brachycephalic dogs

The appearance of the dog is an important factor for owners acquiring a brachycephalic dog, despite health issues linked to the appearance [39, 40]. However, the results from the current study indicate that owning a dog with health issues linked to appearance impacts the owners’ perception of the extent and severity of BOAS-related clinical signs. This is in line with previous research, showing that owning a brachycephalic breed with health issues decreases the likelihood of acquiring another dog of the same breed [40, 41].

The personal experiences of owning/assessing brachycephalic dogs with health issues related to their physical appearance varied both in and between the stakeholder groups. However, the results verify that these health issues are widely spread, as almost all veterinarians and a large fraction of the owners (32.7%) and breeders (24.8%) had personal experiences of dogs affected by these issues. Even though these numbers are not estimates of the prevalence of BOAS, they are supported by the high incidence of upper respiratory tract disorders in French and English bulldogs and pugs reported in a Swedish epidemiologic study [42].

Of the veterinarians that treated brachycephalic dogs during 2019, 94.8% reported that they had diagnosed or treated at least one brachycephalic dog with health issues related to the physical appearance. Of the BSJ who assessed brachycephalic dogs during 2019, 27.4% reported encountering such health issues in the assessed dogs. Veterinarians and show judges might meet different fractions of the same population: veterinarians meet the ones with clinical problems and show judges the ones expected to be healthy representatives of their breed. This also indicates that the most extreme dogs might not reach the show rings.

Besides clinical signs related to breathing and heat regulation, all stakeholders had to a variable extent also experienced health issues related to eyes and skin. Further, the veterinarians indicated that the health issues to a fairly large extent resulted in surgery and even euthanasia. This indicates that the exaggerated anatomical features have a general impact on health and welfare, not only related to BOAS, similar to what is reported in epidemiological papers [43–45], and the Agria Pet Insurance breed profiles for the four focus breeds published on DogWellNet [5].

### Future perspectives

Although this study has shown an agreement between stakeholders on many questions, it has also revealed gaps and disagreements, which highlights the importance of inter-stakeholder collaboration on further measures to improve the health of brachycephalic dogs. Breeders and show judges are key stakeholders, by their selection of breeding stock and by having the possibility and power to reward dogs with healthy conformation and promote breeding with individuals with less exaggerated anatomical features. Veterinarians are key stakeholders by their capacity to recognise clinical signs caused by exaggerated anatomical features. Therefore, it is essential that breeders and BSJ as well as veterinarians share views on the health issues as a basis for further measures, to ensure good health and welfare for future generations of dogs. Breeders and BSJ by acknowledging that too many dogs—even if not seen by them—do suffer from exaggerated BOAS-related anatomical features, requiring measures. Veterinarians by acknowledging that not all dogs with short/wide skulls—even if not seen by them – do suffer from exaggerated BOAS-related anatomic features. That would bridge the gap in perception and facilitate collaborative efforts.

With these disagreements between stakeholders, a multi-stakeholder approach and barriers analysis, aimed to determine the barriers among stakeholders to the desired change [46], would enhance the implementation of feasible strategies to urgently improve the health and welfare issues related to extreme conformation. As concluded by Wolfram et al. (2023) [46], behaviour change can be a challenge even when there is abundant scientific evidence to support the need for a change. Hence, other factors than knowledge or capability are required, such as opportunity and motivation [47]. Multi-stakeholder collaboration is needed [16, 48], in which each stakeholder group contribute by tools in their possession [46], for example by:

- *Veterinary organisations/veterinarians* by their capacity to recognise clinical signs and thereby contribute to a screening of suitable breeding stock.
- *Cynological organisations/breeders* by knowledge about the populations at risk, potential to implement guidelines, and arrange for screening of breeding stock.
- *Show judges* by evaluation of potential breeding stock for anatomical exaggerations that predispose for BOAS.
- *Breeders' organisations* by amending and establishing breed standards and guidelines supporting healthy animals.
- *Authorities* by introducing or amending legislation to support sound breeding and by enforcing existing legislation.
- *Research and academia* by continuing to perform research in e.g. genetics, pathology, and epidemiology, and communicating results to support sound breeding.
- *Animal welfare organisations* by continuing to engage with different stakeholders and pursuing the issue of sound breeding.

### Generalisability and limitations

With dog breeding being international, most findings in our study are likely generalisable to dog populations outside Sweden, although variations in the composition of national populations warrant cautious extrapolation. For example, the Swedish dog population consists mostly of pedigree dogs and relatively few imported dogs [49].

There is a risk of non-response bias, i.e. that the non-responders differ from the responders. There is also a risk of misclassification bias of the stakeholder groups, although this is expected to be low as the groups were defined both according to the email distribution lists and by the responses from the participants. Owners of focus breeds with and without clinical signs related to their appearance were divided based on the owners’ replies. However, it should be noted that clinical signs related to BOAS likely were underestimated by the owners due to the risk of normalisation of clinical signs, as 58% of owners of dogs affected by breathing problems reported that their dog did not have such problems in a previous study [24].

The survey to the veterinarians was distributed in a newsletter from the Swedish Veterinary Association instead of in a separate email as for the other stakeholder groups. This may have contributed to a lower response rate among veterinarians, since it required opening the newsletter in order to see the survey. It is also possible that respondents with experience from dogs with BOAS were more willing to answer to the survey.

## Conclusion

Dog owners and breeders of brachycephalic as well as other breeds, show judges, and veterinarians are well aware of health issues related to the physical appearance of brachycephalic breeds. One-third of the focus breed owners responded that they had owned a dog with health issues related to appearance, verifying the magnitude of BOAS-related health issues in these breeds.

All stakeholders agreed to a great extent that health issues related to conformity threaten the health of brachycephalic breeds and were positive about the fact that the issues are brought to attention. Most were positive to measures that have been taken, and support that guidelines on the appearance of a dog should be based on knowledge about the relationship between health and conformity. However, a disagreement on further measures to be taken and the essentiality of adhering to breed standards was noted, mainly between show judges and veterinarians.

These findings could be used to understand and bridge the gap in opinions between stakeholders and to refine methods to influence the health in dogs with exaggerated brachycephalic features. They also highlight the importance of inter-stakeholder discussions and collaborations, to decrease the occurrence of health issues related to the physical appearance of brachycephalic breeds and promote their welfare.

### Supplementary Information

**Additional file 1: Supplementary Table 1. **An English translated version of a questionnaire regarding health issues related to the appearance of brachycephalic dogs, which was distributed to veterinarians, show judges, dog breeders, and dog owners. **Supplementary Table 2.** Questionnaire responses for the four groups of focus breed owners regarding their awareness and opinions on how to handle health issues related to the physical appearance of brachycephalic breeds. **Supplementary Table 3.** Questionnaire responses for show judges active during 2019 who assessed brachycephalic dogs or not, regarding their awareness and opinions on how to handle of health issues related to the physical appearance of brachycephalic breeds. **Supplementary Table 4.** Questionnaire responses for owners of focus breeds who own/have owned a dog with clinical signs related to the physical appearance and owners of focus breeds without such problems (according to the owner), regarding their awareness and opinions on how to handle of health issues related to the physical appearance of brachycephalic breeds. **Supplementary Table 5.** Questionnaire responses for breeders of a brachycephalic breed and breeders of a non-brachycephalic breed, regarding their awareness and opinions on how to handle health issues related to the physical appearance of brachycephalic breeds.

## Data Availability

The data are available from the authors upon reasonable request.
