# Supplementary material for: Awareness, experiences, and opinions by owners, breeders, show judges, and veterinarians on canine Brachycephalic Obstructive Airway Syndrome (BOAS)
Source: Canine Med Genet. 2024 Mar 8;11:3. doi: 10.1186/s40575-024-00137-4 (PMC10924362; doi:10.1186/s40575-024-00137-4)
Supplement: Supplementary file 1 — Additional file 1: Supplementary Table 1. An English translated version of a questionnaire regarding health issues related to the appearance of brachycephalic dogs, which was distributed to veterinarians, show judges, dog breeders, and dog owners. Supplementary Table 2. Questionnaire responses for the four groups of focus breed owners regarding their awareness and opinions on how to handle health issues related to the physical appearance of brachycephalic breeds. Supplementary Table 3. Questionnaire responses for show judges active during 2019 who assessed brachycephalic dogs or not, regarding their awareness and opinions on how to handle of health issues related to the physical appearance of brachycephalic breeds. Supplementary Table 4. Questionnaire responses for owners of focus breeds who own/have owned a dog with clinical signs related to the physical appearance and owners of focus breeds without such problems (according to the owner), regarding their awareness and opinions on how to handle of health issues related to the physical appearance of brachycephalic breeds. Supplementary Table 5. Questionnaire responses for breeders of a brachycephalic breed and breeders of a non-brachycephalic breed, regarding their awareness and opinions on how to handle health issues related to the physical appearance of brachycephalic breeds. [file 40575_2024_137_MOESM1_ESM.docx]

**Supplementary Table 1.** An English translated version of a questionnaire regarding health issues related to the appearance of brachycephalic dogs, which was distributed to veterinarians, show judges, dog breeders, and dog owners

| *Questions to all participants* |
| --- |
| Q1. I am aware of health issues related to the physical appearance of dogs with short noses   - Yes, a lot - Yes, a little - No |
| Q2. I have seen/met dogs with short noses who had health issues related to their physical appearance   - Yes, several - Yes, one - No - I do not know |
| Q3. My experience is that health issues related to the physical appearance of dogs with short noses have…   - Increased - Not changed - Decreased - I do not know - I do not agree that such health issues exist |
| Q4. I think that health issues related to the physical appearance of short-nosed dogs are a serious threat to their health   - Yes - Partly - No - I do not know |
| Q5. I think it is good that the impact of anatomical features on the health of short-nosed dogs is brought to the attention   - Yes - Partly - No - I do not know - I do not agree that such health issues exist |
| Q6. I think it is positive that measures have been taken to prevent the impact of anatomical features on the health of short-nosed dogs (registration of surgical procedures, guidelines for evaluation at dog shows, certificate of breathing capacity)   - Yes - Partly - No - I do not know |
| Q7. I think further measures should be taken to prevent the impact of anatomical features on the health of short-nosed dogs   - Yes - Partly - No - I do not know |
| Q8. I am positive to a ban on breeding from dogs with such short noses that it increases the risk of health issues (such as breathing problems, impaired regulation of heat, eye injuries, skin problems)   - Yes - Partly - No - I do not know |
| Q9. I am positive to a ban on all breeding of short-nosed dog types or breeds   - Yes - Partly - No - I do not know |
| Q10. I think it is important to follow the breed standard from the breed’s country of origin when choosing breeding animals, even if it can be associated with health issues related to their physical appearance   - Yes - Partly - No - I do not know |
| Q11. I think that guidelines for how short-nosed breeds should look, ought to be decided based on knowledge regarding health issues related to the physical appearance*   - Yes - Partly - No |
| *Question to dog owners* |
| Q12. Have any of your dog(s) showed signs of health issues related to the appearance (consider both currently and previously owned dogs)?   - Yes, many - Yes, one - No - I do not know |
| *Question to dog breeders* |
| Q13. Are you or have you been a breeder of a short-nosed breed?   - Yes - No |
| Q14. Has any of your dog(s) showed signs of health issues related to the appearance?   - Yes, many - Yes, one - No - I do not know |
| *Questions to show judges* |
| Q15. Did you serve as judge during 2019?   - Yes, several times - Yes, one time - No |
| Q16. Did you serve as judge for short-nosed breeds during 2019?   - Yes, several times - Yes, one time - No |
| Q17. Did you at judging come across dogs showing health issues related to the appearance?   - Yes, many - Yes, one - No - I do not know |
| *Questions to veterinarians* |
| Q18. Did you treat any dogs during 2019?   - Yes, several - Yes, one - No |
| Q19. Did you treat any short-nosed dogs during 2019?   - Yes, several - Yes, one - No |
| Q20. Did you diagnose or treat one or several short-nosed dogs with clinical signs related to their appearance in Sweden during 2019?   - Yes, several - Yes, one - No |
| *Questions to dog owners, breeders, show judges and veterinarians who replied yes to Q12, Q14, Q17, or Q20* |
| Q21. Has the dog(s) had affected breathing?   - Yes, many - Yes, one - No - I do not know |
| Q22. Has the dog(s) had an affected ability to regulate heat (i.e. overheating in hot weather)?   - Yes, many - Yes, one - No - I do not know |
| Q23. Has the dog(s) had skin or eye problems?   - Yes, many - Yes, one - No - I do not know |
| Q24. Were the health issues surgically treated?   - Yes, in several dogs - Yes, in one dog - No - I do not know |
| Q25. Did the health issues result in death/euthanasia?   - Yes, for several dogs - Yes, for one dog - No - I do not know |

**Supplementary Table 2.** Questionnaire responses for the four groups of focus breed owners regarding their awareness and opinions on how to handle health issues related to the physical appearance of brachycephalic breeds

|  | Boston terrier  (n = 154) | English bulldog (n = 85) | French bulldog  (n = 109) | Pug  (n = 117) |
| --- | --- | --- | --- | --- |
| Q1. I am aware of health issues related to the physical appearance of dogs with short noses | | | | |
| Yes, a lot | 118 (76.6%) | 65 (76.5%) | 90 (82.6%) | 94 (80.3%) |
| Yes, a little | 34 (22.1%) | 19 (22.4%) | 19 (17.4%) | 23 (19.7%) |
| No | 2 (1.30%) | 1 (1.18%) | 0 (0%) | 0 (0%) |
| Q2. I have seen/met dogs with a short nose who have health issues related to how they look* | | | | |
| Yes, several | 69 (44.8%) | 28 (32.9%) | 65 (59.6%) | 41 (35.0%) |
| Yes, one | 26 (16.9%) | 18 (21.2%) | 22 (20.2%) | 22 (18.8%) |
| No | 39 (25.3%) | 29 (34.1%) | 13 (11.9%) | 45 (38.5%) |
| I do not know | 20 (13.0%) | 10 (11.8%) | 9 (8.26%) | 9 (7.69%) |
| Q3. My experience is that health issues related to the physical appearance of dogs with short noses have… | | | | |
| Increased | 15 (9.74%) | 10 (11.8%) | 14 (12.8%) | 12 (10.3%) |
| No change | 41 (26.6%) | 28 (32.9%) | 30 (27.5%) | 34 (29.1%) |
| Decreased | 26 (16.9%) | 15 (17.6%) | 14 (12.8%) | 13 (11.1%) |
| I do not know | 70 (45.5%) | 30 (35.3%) | 51 (46.8%) | 57 (48.7%) |
| I do not agree that such health issues exist | 2 (1.30%) | 2 (2.35%) | 0 (0%) | 1 (0.85%) |
| Q4. I think that health issues related to the physical appearance of short-nosed dogs are a serious threat to their health* | | | | |
| Yes | 48 (31.2%) | 16 (18.8%) | 43 (39.4%) | 26 (22.2%) |
| Partly | 77 (50.0%) | 41 (48.2%) | 49 (45.0%) | 54 (46.2%) |
| No | 13 (8.44%) | 14 (16.5%) | 7 (6.42%) | 25 (21.4%) |
| I do not know | 16 (10.4%) | 14 (16.5%) | 10 (9.17%) | 12 (10.3%) |
| Q5 I think it is good that the impact of the anatomical features of short-nosed dogs on their health is being brought to attention | | | | |
| Yes | 117 (76.0%) | 55 (64.7%) | 81 (74.3%) | 69 (59.0%) |
| Partly | 25 (16.2%) | 21 (24.7%) | 20 (18.3%) | 31 (26.5%) |
| No | 1 (0.65%) | 1 (1.18%) | 2 (1.83%) | 3 (2.56%) |
| I do not know | 4 (2.60%) | 4 (4.71%) | 3 (2.75%) | 7 (5.98%) |
| I do not agree that such health issues exist | 7 (4.55%) | 4 (4.71%) | 3 (2.75%) | 7 (5.98%) |
| Q6. I think it is positive that measures have been taken to prevent the impact of anatomical features on the health of short-nosed dogs (registration of surgical procedures, guidelines for evaluation at dog shows, certificate of breathing capacity) | | | | |
| Yes | 127 (82.5%) | 58 (68.2%) | 83 (76.1%) | 86 (73.5%) |
| Partly | 16 (10.4%) | 15 (17.6%) | 16 (14.7%) | 16 (13.7%) |
| No | 2 (1.30%) | 1 (1.18%) | 3 (2.75%) | 7 (5.98%) |
| I do not know | 9 (5.84%) | 11 (12.9%) | 7 (6.42%) | 8 (6.84%) |
| Q7. I think further measures should be taken to prevent the impact of anatomical features on the health of short-nosed dogs* | | | | |
| Yes | 75 (48.7%) | 32 (37.6%) | 61 (56.0%) | 43 (36.8%) |
| Partly | 40 (26.0%) | 27 (31.8%) | 31 (28.4%) | 31 (26.5%) |
| No | 15 (9.74%) | 11 (12.9%) | 5 (4.59%) | 18 (15.4%) |
| I do not know | 24 (15.6%) | 15 (17.6%) | 12 (11.0%) | 25 (21.4%) |
| Q8. I am positive to a ban on breeding from dogs with such short noses that it increases the risk of health issues (such as breathing problems, impaired regulation of heat, eye injuries, skin problems)* | | | | |
| Yes | 92 (59.7%) | 46 (54.1%) | 67 (61.5%) | 45 (38.5%) |
| Partly | 36 (23.4%) | 12 (14.1%) | 20 (18.3%) | 31 (26.5%) |
| No | 20 (13.0%) | 20 (23.5%) | 17 (15.6%) | 34 (29.1%) |
| I do not know | 6 (3.90%) | 7 (8.23%) | 5 (4.59%) | 7 (5.98%) |
| Q9. I am positive to a ban on all breeding of short-nosed dog types or breeds | | | | |
| Yes | 4 (2.60%) | 6 (7.06%) | 8 (7.34%) | 6 (5.13%) |
| Partly | 17 (11.0%) | 9 (10.6%) | 13 (11.9%) | 13 (11.1%) |
| No | 127 (82.5%) | 68 (80.0%) | 81 (74.3%) | 88 (75.2%) |
| I do not know | 6 (3.90%) | 2 (2.35%) | 7 (6.42%) | 10 (8.55%) |
| Q10. I think it is important to follow the breed standard from the breed’s country of origin when choosing breeding animals, even if it can be associated with health issues related to their physical appearance* | | | | |
| Yes | 25 (16.2%) | 10 (11.8%) | 18 (16.5%) | 14 (12.0%) |
| Partly | 29 (18.8%) | 26 (30.6%) | 14 (12.8%) | 20 (17.1%) |
| No | 83 (53.9%) | 39 (45.9%) | 60 (55.0%) | 57 (48.7%) |
| I do not know | 17 (11.0%) | 10 (11.8%) | 17 (15.6%) | 26 (22.2%) |
| Q11. I think that guidelines for how short-nosed breeds should look, ought to be decided based on knowledge regarding health issues related to the physical appearance | | | | |
| Yes | 121 (78.6%) | 64 (75.3%) | 89 (81.7%) | 82 (70.1%) |
| Partly | 29 (18.8%) | 17 (20.0%) | 16 (14.7%) | 29 (24.8%) |
| No | 4 (2.60%) | 4 (4.71%) | 4 (3.67%) | 6 (5.13%) |

*P < 0.05 for intergroup comparisons (Chi-squared test or Fisher’s exact test)

**Supplementary Table 3.** Questionnaire responses for show judges active during 2019 who assessed brachycephalic dogs or not, regarding their awareness and opinions on how to handle of health issues related to the physical appearance of brachycephalic breeds

|  | Show judge, brachycephalic breeds (n = 73) | Show judge, other breeds  (n = 45) |
| --- | --- | --- |
| Q1. I am aware of health issues related to the physical appearance of dogs with short noses | | |
| Yes, a lot | 63 (86.3%) | 37 (82.2%) |
| Yes, a little | 8 (13.7%) | 10 (17.8%) |
| No | 0 (0%) | 0 (0%) |
| Q2. I have seen/met dogs with a short nose who have health issues related to how they look | | |
| Yes, several | 40 (54.8%) | 28 (62.2%) |
| Yes, one | 15 (20.5%) | 6 (17.8%) |
| No | 18 (24.7%) | 18 (24.7%) |
| I do not know | 0 (0%) | 3 (6.7%) |
| Q3. My experience is that health issues related to the physical appearance of dogs with short noses have…* | | |
| Increased | 8 (11.0%) | 16 (35.6%) |
| No change | 30 (41.1%) | 11 (24.4%) |
| Decreased | 29 (39.7%) | 9 (20.0%) |
| I do not know | 4 (5.5%) | 8 (17.8%) |
| I do not agree that such health issues exist | 2 (2.7%) | 1 (2.2%) |
| Q4. I think that health issues related to the physical appearance of short-nosed dogs are a serious threat to their health* | | |
| Yes | 11 (15.1%) | 20 (44.4%) |
| Partly | 41 (56.2%) | 18 (40.0%) |
| No | 20 (27.4%) | 6 (11.1%) |
| I do not know | 1 (1.4%) | 2 (4.4%) |
| Q5. I think it is good that the impact of anatomical features on the health of short-nosed dogs is brought to the attention* | | |
| Yes | 28 (38.4%) | 30 (66.7%) |
| Partly | 33 (45.2%) | 12 (26.7%) |
| No | 3 (4.1%) | 1 (2.2%) |
| I do not agree that such health issues exist | 7 (9.6%) | 1 (2.2%) |
| I do not know | 1 (4.1%) | 1 (2.2%) |
| Q6. I think it is positive that measures have been taken to prevent the impact of anatomical features on the health of short-nosed dogs (registration of surgical procedures, guidelines for evaluation at dog shows, certificate of breathing capacity)* | | |
| Yes | 36 (49.3%) | 34 (75.6%) |
| Partly | 29 (39.7%) | 29 (20.0%) |
| No | 7 (9.6%) | 1 (2.2%) |
| I do not know | 1 (1.4%) | 1 (2.2%) |
| Q7. I think further measures should be taken to prevent the impact of anatomical features on the health of short-nosed dogs* | | |
| Yes | 9 (12.3%) | 13 (28.9%) |
| Partly | 20 (27.4%) | 16 (35.6%) |
| No | 38 (52.1%) | 9 (20.0%) |
| I do not know | 6 (8.2%) | 7 (15.6%) |
| Q8. I am positive to a ban on breeding from dogs with such short noses that it increases the risk of health issues (such as breathing problems, impaired regulation of heat, eye injuries, skin problems)* | | |
| Yes | 6 (8.2%) | 21 (46.7%) |
| Partly | 18 (24.7%) | 10 (22.2%) |
| No | 46 (63.0%) | 12 (26.7%) |
| I do not know | 3 (4.1%) | 2 (4.4%) |
| Q9. I am positive to a ban on all breeding of short-nosed dog types or breeds | | |
| Yes | 0 (0%) | 1 (2.2%) |
| Partly | 4 (5.5%) | 5 (11.1%) |
| No | 69 (94.5%) | 37 (82.2%) |
| I do not know | 0 (0%) | 2 (4.4%) |
| Q10. I think it is important to follow the breed standard from the breed’s country of origin when choosing breeding animals, even if it can be associated with health issues related to their physical appearance* | | |
| Yes | 27 (37.0%) | 6 (13.3%) |
| Partly | 15 (20.5%) | 22 (48.9%) |
| No | 26 (35.6%) | 12 (26.7%) |
| I do not know | 5 (6.8%) | 5 (11.1%) |
| Q11. I think that guidelines for how short-nosed breeds should look, ought to be decided based on knowledge regarding health issues related to the physical appearance* | | |
| Yes | 30 (41.1%) | 25 (55.6%) |
| Partly | 33 (45.2%) | 20 (44.4%) |
| No | 10 (13.7%) | 0 (0%) |

*P < 0.05 for intergroup comparisons (Chi-squared test or Fisher’s exact test)

**Supplementary Table 4.** Questionnaire responses for owners of brachycephalic dogs who own/have owned a dog with clinical signs related to the physical appearance and owners of brachycephalic dogs without such problems (according to the owner), regarding their awareness and opinions on how to handle of health issues related to the physical appearance of brachycephalic breeds

|  | Owner of a brachycephalic dog with clinical signs (n = 152) | Owner of a brachycephalic dog without clinical signs (n = 313) |
| --- | --- | --- |
| Q1. I am aware of health issues related to the physical appearance of dogs with short noses* | | |
| Yes, a lot | 137 (90.1%) | 230 (73.5%) |
| Yes, a little | 15 (9.87%) | 80 (25.6%) |
| No | 0 (0%) | 3 (0.96%) |
| Q2. I have seen/met dogs with a short nose who have health issues related to how they look* | | |
| Yes, several | 104 (68.4%) | 99 (31.6%) |
| Yes, one | 28 (18.4%) | 60 (19.2%) |
| No | 10 (6.58%) | 116 (37.1%) |
| I do not know | 10 (6.58%) | 38 (12.1%) |
| Q3. My experience is that health issues related to the physical appearance of dogs with short noses have…* | | |
| Increased | 27 (17.8%) | 24 (7.67%) |
| No change | 46 (30.3%) | 87 (27.8%) |
| Decreased | 17 (11.2%) | 51 (16.3%) |
| I do not know | 62 (40.8%) | 146 (46.6%) |
| I do not agree that such health issues exist | 0 (0%) | 5 (1.60%) |
| Q4. I think that health issues related to the physical appearance of short-nosed dogs are a serious threat to their health* | | |
| Yes | 64 (42.1%) | 69 (22.0%) |
| Partly | 73 (48.0%) | 148 (47.3%) |
| No | 7 (4.61%) | 52 (16.6%) |
| I do not know | 8 (5.26%) | 44 (14.1%) |
| Q5. I think it is good that the impact of anatomical features on the health of short-nosed dogs is brought to the attention* | | |
| Yes | 128 (84.2%) | 194 (62.0%) |
| Partly | 17 (11.2%) | 80 (25.6%) |
| No | 2 (1.32%) | 5 (1.60%) |
| I do not agree that such health issues exist | 2 (1.32%) | 19 (6.07%) |
| I do not know | 3 (1.97%) | 15 (4.79%) |
| Q6. I think it is positive that measures have been taken to prevent the impact of anatomical features on the health of short-nosed dogs (registration of surgical procedures, guidelines for evaluation at dog shows, certificate of breathing capacity)* | | |
| Yes | 131 (86.2%) | 223 (71.2%) |
| Partly | 15 (9.87%) | 48 (15.3%) |
| No | 0 (0%) | 13 (4.15%) |
| I do not know | 6 (3.95%) | 29 (9.27%) |
| Q7. I think further measures should be taken to prevent the impact of anatomical features on the health of short-nosed dogs* | | |
| Yes | 96 (63.2%) | 115 (36.7%) |
| Partly | 32 (21.1%) | 97 (31.0%) |
| No | 7 (4.61%) | 42 (13.4%) |
| I do not know | 17 (11.2%) | 59 (18.8%) |
| Q8. I am positive to a ban on breeding from dogs with such short noses that it increases the risk of health issues (such as breathing problems, impaired regulation of heat, eye injuries, skin problems)* | | |
| Yes | 96 (63.2%) | 154 (49.2%) |
| Partly | 38 (25.0%) | 61 (19.5%) |
| No | 14 (9.21%) | 77 (24.6%) |
| I do not know | 4 (2.63%) | 21 (6.71%) |
| Q9. I am positive to a ban on all breeding of short-nosed dog types or breeds* | | |
| Yes | 16 (10.5%) | 8 (2.56%) |
| Partly | 27 (17.8%) | 25 (7.99%) |
| No | 104 (68.4%) | 260 (83.1%) |
| I do not know | 5 (3.29%) | 20 (6.39%) |
| Q10. I think it is important to follow the breed standard from the breed’s country of origin when choosing breeding animals, even if it can be associated with health issues related to their physical appearance | | |
| Yes | 22 (14.5%) | 45 (14.4%) |
| Partly | 30 (19.7%) | 59 (18.8%) |
| No | 84 (55.3%) | 155 (49.5%) |
| I do not know | 1. (10.5%) | 54 (17.3%) |
| Q11. I think that guidelines for how short-nosed breeds should look, ought to be decided based on knowledge regarding health issues related to the physical appearance* | | |
| Yes | 133 (87.5%) | 223 (71.2%) |
| Partly | 18 (11.8%) | 73 (23.3%) |
| No | 1 (0.66%) | 17 (5.43%) |

*P < 0.05 for intergroup comparisons (Chi-squared test or Fisher’s exact test)

**Supplementary Table 5.** Questionnaire responses for breeders of a brachycephalic breed and breeders of a non-brachycephalic breed, regarding their awareness and opinions on how to handle health issues related to the physical appearance of brachycephalic breeds

|  | Breeder of a brachycephalic breed (n = 137) | Breeder of a non-brachycephalic breed (n = 1414) |
| --- | --- | --- |
| Q1. I am aware of health issues related to the physical appearance of dogs with short noses | | |
| Yes, a lot | 122 (89.1%) | 1184 (83.7%) |
| Yes, a little | 15 (10.9%) | 214 (15.1%) |
| No | 0 (0%) | 16 (1.1%) |
| Q2. I have seen/met dogs with a short nose who have health issues related to how they look * | | |
| Yes, several | 77 (56.2%) | 951 (67.2%) |
| Yes, one | 20 (14.6%) | 169 (12.0%) |
| No | 33 (24.1%) | 204 (14.4%) |
| Don’t know | 7 (5.1%) | 90 (6.4%) |
| Q3. My experience is that health issues related to the physical appearance of dogs with short noses have…* | | |
| Increased | 12 (8.8%) | 589 (41.7%) |
| No change | 62 (45.3%) | 347 (24.5%) |
| Decreased | 34 (24.8%) | 113 (8.0%) |
| Don’t know | 29 (21.2%) | 356 (25.2%) |
| Don’t exist | 0 (0%) | 9 (0.6%) |
| Q4. I think that health issues related to the physical appearance of short-nosed dogs are a serious threat to their health* | | |
| Yes | 28 (20.4%) | 921 (65.1%) |
| Partly | 75 (54.7%) | 353 (25.0%) |
| No | 28 (20.4%) | 65 (4.6%) |
| Don’t know | 6 (4.4%) | 75 (5.3%) |
| Q5. I think it is good that the impact of anatomical features on the health of short-nosed dogs is brought to the attention* | | |
| Yes | 63 (46.0%) | 1190 (84.2%) |
| Partly | 163 (11.5%) | 163 (11.5%) |
| No | 13 (0.9%) | 13 (0.9%) |
| Don’t exist | 12 (8.8%) | 25 (1.8%) |
| Don’t know | 1 (0.7%) | 23 (1.6%) |
| Q6. I think it is positive that measures have been taken to prevent the impact of anatomical features on the health of short-nosed dogs (registration of surgical procedures, guidelines for evaluation at dog shows, certificate of breathing capacity)* | | |
| Yes | 83 (60.6%) | 1188 (84.0%) |
| Partly | 39 (28.5%) | 154 (10.9%) |
| No | 10 (7.3%) | 33 (2.3%) |
| Don’t know | 5 (3.6%) | 39 (2.8%) |
| Q7. I think further measures should be taken to prevent the impact of anatomical features on the health of short-nosed dogs* | | |
| Yes | 36 (26.3%) | 840 (59.4%) |
| Partly | 43 (31.4%) | 276 (19.5%) |
| No | 49 (35.8%) | 122 (8.6%) |
| Don’t know | 9 (6.6%) | 176 (12.4%) |
| Q8. I am positive to a ban on breeding from dogs with such short noses that it increases the risk of health issues (such as breathing problems, impaired regulation of heat, eye injuries, skin problems) | | |
| Yes | 36 (26.3%) | 981 (69.4%) |
| Partly | 39 (28.5%) | 255 (18.0%) |
| No | 59 (43.1%) | 135 (9.5%) |
| Don’t know | 3 (2.2%) | 43 (3.0%) |
| Q9. I am positive to a ban on all breeding of short-nosed dog types or breeds* | | |
| Yes | 0 (0%) | 229 (16.2%) |
| Partly | 10 (7.3%) | 325 (23.0%) |
| No | 125 (91.2%) | 774 (54.7%) |
| Don’t know | 2 (1.5%) | 86 (6.1%) |
| Q10. I think it is important to follow the breed standard from the breed’s country of origin when choosing breeding animals, even if it can be associated with health issues related to their physical appearance* | | |
| Yes | 34 (24.8%) | 223 (15.8%) |
| Partly | 43 (31.4%) | 288 (20.4%) |
| No | 57 (41.6%) | 804 (56.9%) |
| Don’t know | 3 (2.2%) | 99 (7.0%) |
| Q11. I think that guidelines for how short-nosed breeds should look, ought to be decided based on knowledge regarding health issues related to the physical appearance* | | |
| Yes | 80 (58.4%) | 1192 (84.3%) |
| Partly | 44 (32.1%) | 196 (13.9%) |
| No | 13 (9.5%) | 26 (1.8%) |

*P < 0.05 for intergroup comparisons (Chi-squared test or Fisher’s exact test)
